# Bma-LAD-2, an Intestinal Cell Adhesion Protein, as a Potential Therapeutic Target for Lymphatic Filariasis

**DOI:** 10.1128/mbio.03742-21

**Published:** 2022-04-27

**Authors:** Alexander F. Flynn, Rebekah T. Taylor, Marzena E. Pazgier, Sasisekhar Bennuru, Alyssa R. Lindrose, Spencer L. Sterling, C. Paul Morris, Brynna I. Gleeson, Tim K. Maugel, Thomas B. Nutman, Edward Mitre

**Affiliations:** a Department of Microbiology, F. Edward Hébert School of Medicine, Uniformed Services University of the Health Sciences, Bethesda, Maryland, USA; b Department of Biology, Frostburg State University, Frostburg, Maryland, USA; c Department of Medicine, F. Edward Hébert School of Medicine, Uniformed Services University of the Health Sciences, Bethesda, Maryland, USA; d National Institute of Allergy and Infectious Disease, National Institutes of Health, Bethesda, Maryland, USA; e The Henry M. Jackson Foundation for the Advancement of Military Medicine, Inc., Bethesda, Maryland, USA; f Department of Pathology, Johns Hopkins Hospital, Baltimore, Maryland, USA; g Laboratory for Biological Ultrastructure, Department of Biology, University of Maryland, Silver Spring, Maryland, USA; Albert Einstein College of Medicine

**Keywords:** *Brugia malayi*, *Brugia pahangi*, *Brugia timori*, neglected tropical diseases, *Wuchereria bancrofti*, filaria, filariasis, helminth, siRNA, vaccine

## Abstract

Lymphatic filariasis is a debilitating disease that afflicts over 70 million people worldwide. It is caused by the parasitic nematodes Wuchereria bancrofti, Brugia malayi, and Brugia timori. Despite substantial success, efforts to eliminate LF will likely require more time and resources than predicted. Identifying new drug and vaccine targets in adult filariae could help elimination efforts. This study’s aim was to evaluate intestinal proteins in adult Brugia malayi worms as possible therapeutic targets. Using short interfering RNA (siRNA), we successfully targeted four candidate gene transcripts: Bma-Serpin, Bma-ShTK, Bma-Reprolysin, and Bma-LAD-2. Of those, Bma-LAD-2, an immunoglobulin superfamily cell adhesion molecule (IgSF CAM), was determined to be essential for adult worm survival. We observed a 70.42% knockdown in Bma-LAD-2 transcript levels 1 day post-siRNA incubation and an 87.02% reduction in protein expression 2 days post-siRNA incubation. This inhibition of Bma-LAD-2 expression resulted in an 80% decrease in worm motility over 6 days, a 93.43% reduction in microfilaria release (Mf) by day 6 post-siRNA incubation, and a dramatic decrease in (4,5-dimethylthiazol-2-yl)-2,5-diphenyltetrazolium bromide (MTT) reduction. Transmission electron microscopy revealed the loss of microvilli and unraveling of mitochondrial cristae in the intestinal epithelium of Bma-LAD-2 siRNA-treated worms. Strikingly, Bma-LAD-2 siRNA-treated worms exhibited an almost complete loss of pseudocoelomic fluid. A luciferase immunoprecipitation system assay did not detect anti-Bma-LAD-2 IgE in the serum of 30 LF patients, indicating that LF exposure does not result in IgE sensitization to this antigen. These results indicate that Bma-LAD-2 is an essential protein for adult Brugia malayi and may be an effective therapeutic target.

## INTRODUCTION

Over 70 million people are infected worldwide with lymphatic filariasis (LF) (1), a debilitating disease characterized by severe lymphedema, elephantiasis, and hydrocele (2, 3). LF is caused by the parasitic nematodes Wuchereria bancrofti, Brugia malayi, and Brugia timori. Currently, efforts to eliminate this disease have been spearheaded by the Global Program to Eliminate Lymphatic Filariasis (GPELF) (4). While this campaign has reduced the overall prevalence of the disease, elimination target dates have been difficult to meet. According to a January 2020 WHO report on ending neglected tropical diseases, of the 71 countries where LF was endemic in 2000, only 17 have been declared free of LF as a public health problem. The original goal set by the GPELF called for global elimination of LF as a public health problem by 2020, but this WHO report established a new goal of eliminating LF as a public health problem from 81% of countries where it is endemic by 2030 (5). New strategies and therapeutics would likely improve our ability to meet this new target (6–8).

Current therapies for LF include diethylcarbamazine (DEC), ivermectin (IVM), and albendazole. While triple-drug therapy with all three of these agents has shown great promise (9, 10), a major limitation of these medications is that DEC and IVM cannot be administered empirically in areas endemic for *Loa loa* or Onchocerca volvulus because the drugs can precipitate severe side effects by rapid killing of Mf (11–14).

To avoid side effects from killing of microfilariae (Mf) in coendemic populations and to potentially enable a single treatment cure of filarial infections, our group has focused on identifying drug and/or vaccine targets specific to adult filarial worms. Because adult worms contain a complete intestinal tract, whereas microfilariae do not, our group evaluated the intestinal tract of adult filarial worms as a possible source of therapeutic targets. Already, this strategy appears to be promising against other helminths. Numerous studies have demonstrated protection against hookworm and barber pole worm infection using nematode intestinal antigens as vaccine candidates (15–20). Furthermore, there seems to be little specific IgE against intestinal antigens in the sera of infected animal models as well as in previously exposed individuals (21, 22), suggesting that intestinal antigens are safe to administer as vaccines in areas where the disease is endemic.

Our lab previously performed a proteomic analysis of the body wall, gut, and reproductive tract of *Brugia* adult worms (23). We identified 396 proteins specific for the intestine and then selected 9 for evaluation as potential drug and therapeutic targets. The selection criteria were having (i) high homology with orthologs in other filarial species and low homology to humans, (ii) a large extracellular domain potentially accessible to drugs and antibody, and (iii) a predicted function likely essential for adult filarial survival. Previous work we have conducted found that a filarial intestinal antigen, Bm-UGT (UDP-glucuronosyl transferase), was essential for adult B. malayi survival and could be targeted with probenecid to achieve death of adult worms (24).

Using short interfering RNA (siRNA) inhibition, we successfully knocked down 4 target proteins. Of these, Bma-LAD-2, an immunoglobulin superfamily cell adhesion molecule (IgSF CAM), was found to be essential for adult worm survival. Suppression of Bma-LAD-2 expression resulted in decreased worm motility, metabolism, and Mf release. Electron microscopy revealed that inhibition of Bma-LAD-2 resulted in almost complete loss of pseudocoelomic fluid, suggesting that disrupting the tight junctions between filarial intestinal cells and causing subsequent disruption of the worms’ hydrostatic skeleton is a novel mechanism to kill filarial parasites.

## RESULTS

### Structural analysis of Bma-LAD-2.

**FIG 1:**
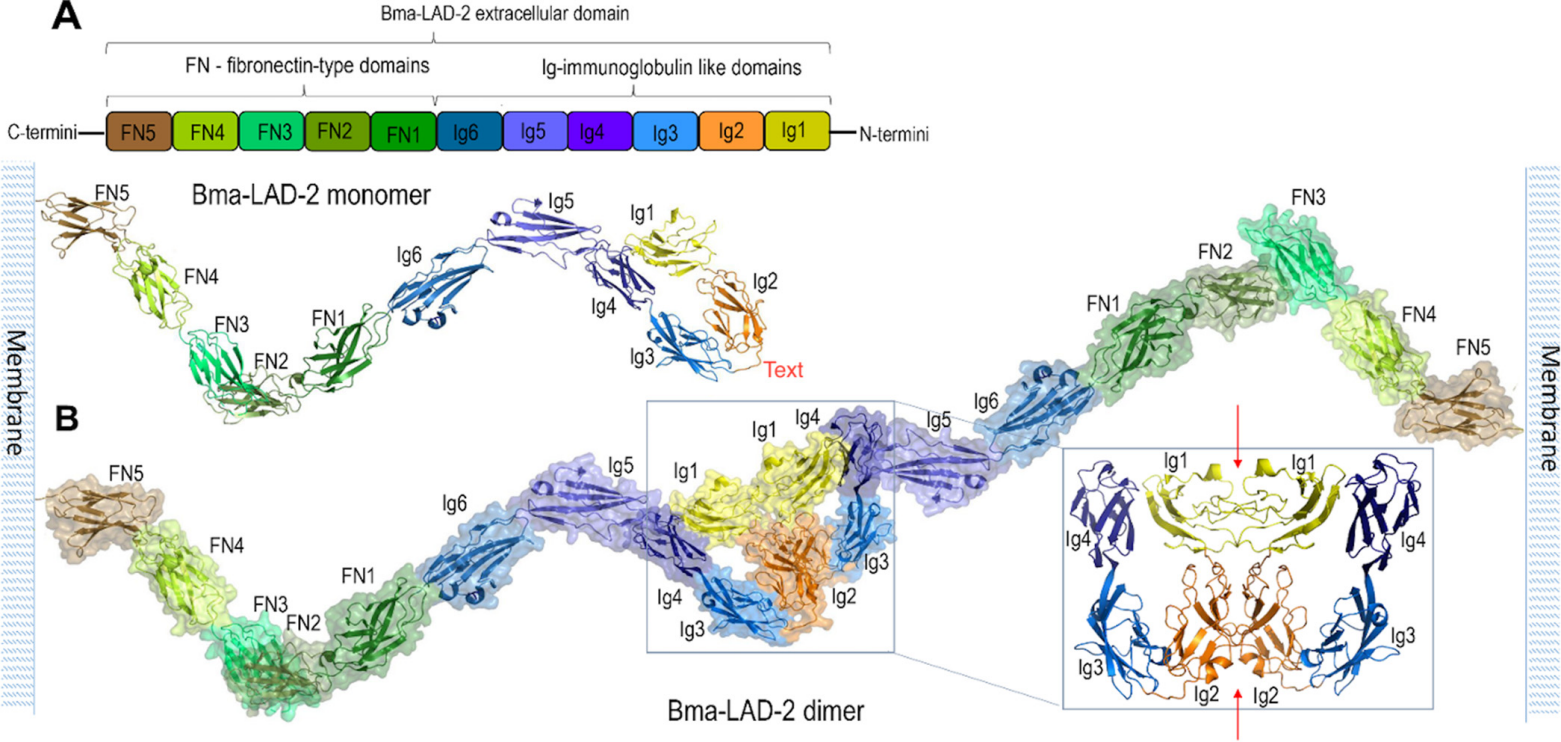
Molecular organization of Bma-LAD-2 extracellular domain. (A) Bma-LAD-2 monomer. Schematic domain organization (top) and model of monomer structure (bottom) assembled based on sequence similarity and available crystal structures of homologous proteins as described in Materials and Methods. (B) Putative structure of Bma-LAD-2 dimer. Expanded view shows the dimer interface (indicated by red arrows) with Ig domains as labeled.

The Bma-LAD-2 protein is 1,171 amino acids (aa) in length (molecular mass of 133,310.4 Da), with a signal peptide, aa 1 to 18, a large extracellular segment at position 19 to 1120, a transmembrane portion at aa 1121 to 1143, and a small cytoplasmic domain at position 1143 to 1171 (see Fig. S1 in the supplemental material). The putative domain organization and model of the structure of the extracellular domain (residues 18 to 1120) is shown in Fig. 1 for both the Bma-LAD-2 monomer and dimer. The Bma-LAD-2 monomer is predicted to fold into 6 immunoglobulin domains (Ig1-Ig6) followed by 5 fibronectin-type domains (FN1 to -5) (Fig. 1A). The outermost N-terminal Ig domains are predicted to homodimerize to form tight junctions. The Bma-LAD-2 dimer model, based on dimerization mode of the homologous protein neurofascin (25), is stabilized by contacts between the domains of Ig1 and Ig2 paired in an orthogonal side-to-side stacking mode (Fig. 1B).

10.1128/mbio.03742-21.2 FIG S1 Amino acid sequence for Bma-LAD-2. Highlighted sections correspond to the molecular domains of the protein. Download FIG S1, JPG file, 1.2 MB. Copyright © 2022 Flynn et al. 2022 Flynn et al. https://creativecommons.org/licenses/by/4.0/ This content is distributed under the terms of the Creative Commons Attribution 4.0 International license.

Neurofascin is a member of the immunoglobulin gene superfamily, which includes other neural cell adhesion molecules such as mammalian neural cell adhesion molecule L1 (CD171) and their homologs in other species. All immunoglobulin superfamily proteins contain tandem Ig-like domains followed by, in some cases, fibronectin type III (FNIII) domains. (26, 27). Structure-function analyses indicate that a horseshoe-shaped dimer formed by four N-terminal Ig-like domains in neurofascin and other cell adhesion immunoglobulin superfamily members is required for their adhesive function (25, 27, 28).

It is likely that prevention and/or disruption of formation of the tight Ig junction or destabilization and/or disruption of the Bma-LAD-2 dimer lead to loss of Bma-LAD-2 function.

### Bma-LAD-2 is phylogenetically related to orthologs found in other filarial worms.

**FIG 2:**
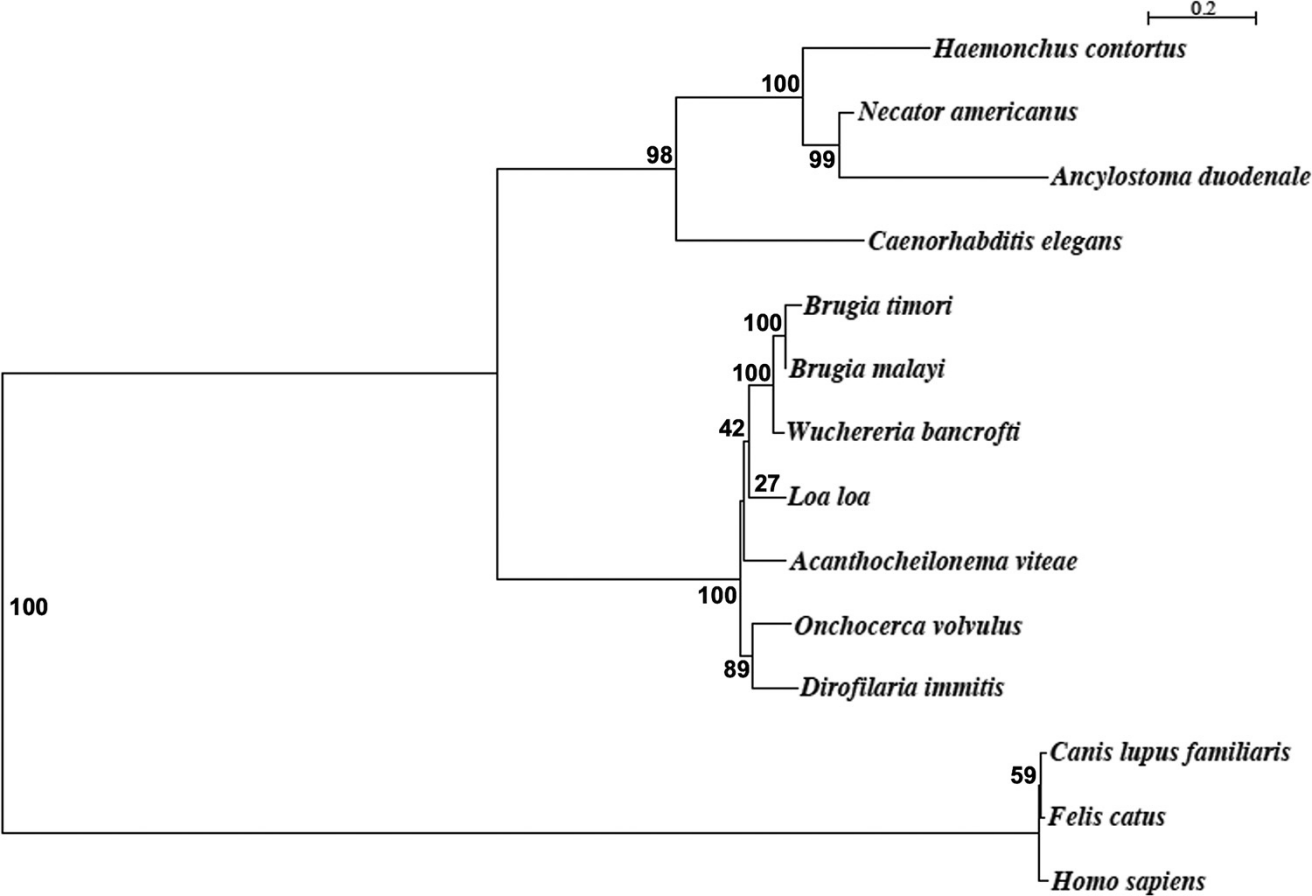
Phylogenetic tree of *Bma-*LAD-2 and orthologs from other helminths. The amino acid sequence of *Bma*-LAD-2 (WormBase gene ID WBGene00227085) has high level of relatedness to other filarial species and is evolutionarily distant from cats, dogs, and humans. Based on sequence alignment using multiple-sequence comparison by log expectation (MUSCLE), the phylogenetic tree was constructed by the maximum likelihood method. The phylogenetic scale represents genetic change as defined by the average number of nucleotide substitutions per site. The numbers at each branch represent the bootstrap value out of 100.

Bma-LAD-2 was previously shown to be a protein localized to the gut of adult B. malayi worms and to have a high predicted sequence homology with other filarial orthologs (23). In this study, we generated a phylogenetic tree (Fig. 2) to view the level of evolutionary relatedness between Bma-LAD-2 and orthologs from other filarial species and helminths. We found a close phylogenetic relation between Bma-LAD-2 and orthologs found in other filarial species and with orthologs of the intestinal helminths. Furthermore, the large phylogenetic distance to orthologs in humans, dogs, and cats suggests that filarial protein can be targeted by medications or vaccines with little risk to the host.

### Bma-LAD-2 is expressed throughout the life cycle of B. malayi adult worms.

To determine whether Bma-LAD-2 is expressed in a stage-specific manner, we analyzed stage-specific transcriptomic data on Brugia malayi worms (29). We found that Bma-LAD-2 RNA was expressed in the Mf, L3, L4, and adult stages regardless of gender. The highest expression levels based on normalized read values occurred in mature microfilaria (44 reads per kilobase per million [RPKM]) followed by adult female filaria (26 RPKM). Overall, Bma-LAD-2 transcript levels appeared to be similar across the different life stages. Next, we looked at a study evaluating the proteome for the different stages of B. malayi (30). The study matched 3 unique peptides to Bma-LAD-2 from Mf, 1 from the L3 stage, 2 from adult females, and 1 from adult males, suggesting that Bma-LAD-2 is expressed during Mf and L3 stages as well as in both genders of adult worms. Like the transcriptome data, these spectrum values indicate fairly consistent expression of Bma-LAD-2 among the larval stages.

### cy3-labeled siRNA enters the intestinal tract of B. malayi adult worms.

**FIG 3:**
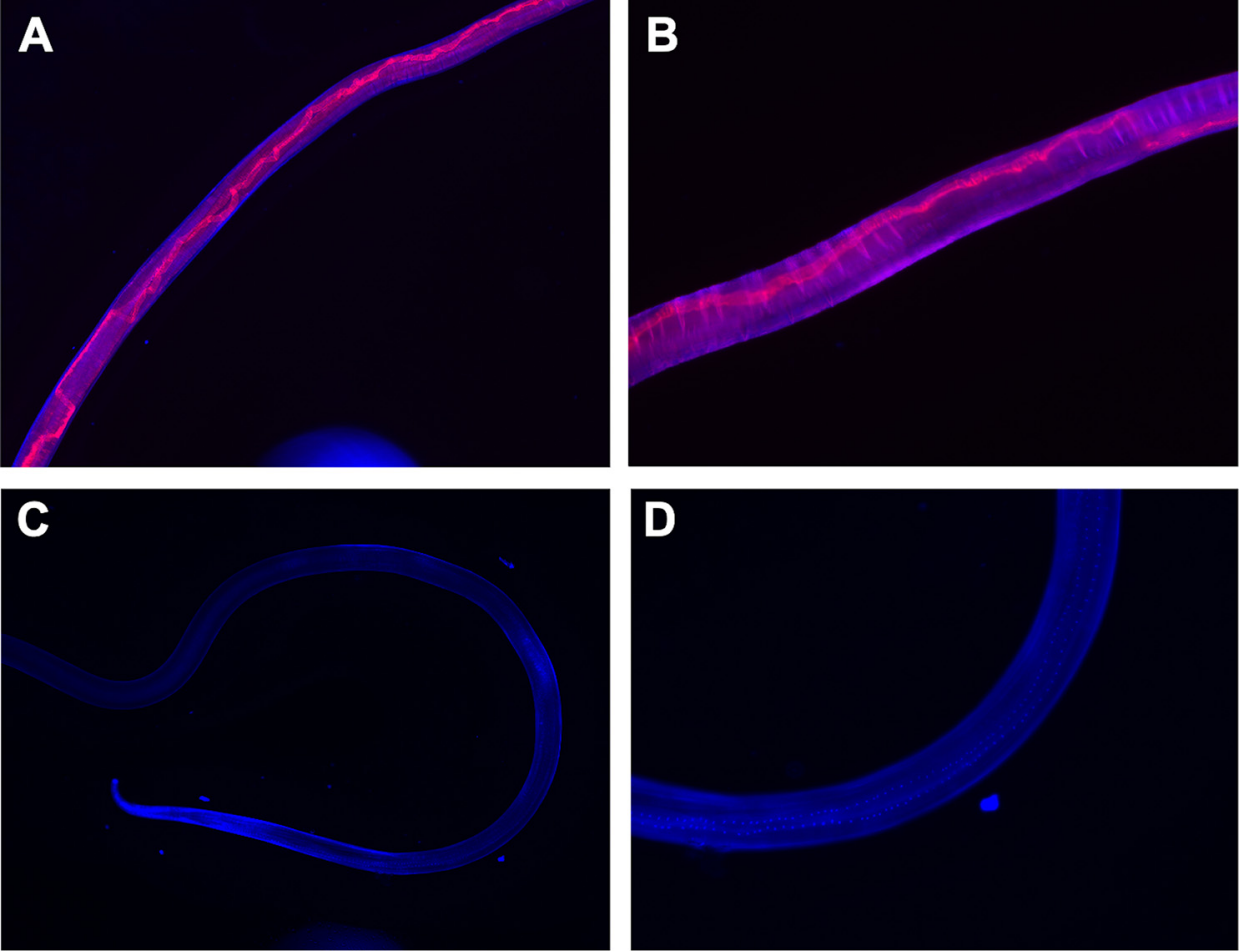
cy3-labeled *Bma*-LAD-2 siRNA entry into the intestinal tract of female B. malayi adult worms. B. malayi adult female worms soaked in cy3-labeled siRNA (red) for 24 h and visualized at magnifications of 40× (A) and 100× (B). As a negative control, worms were cultured in medium only for 24 h and visualized at magnifications of 40× (C) and 100× (D). Worms were counterstained with DAPI (blue).

Prior to siRNA knockdown, we investigated whether Bma-LAD-2 siRNA conjugated to cy3 could be visualized in the intestinal tract of adult filariae. We incubated the adult female worms with labeled siRNA for 24 h and then observed them using epifluorescence microscopy. The cy3-labeled siRNA (Fig. 3A and B) was easily seen in the intestinal tract of the treated worms. As expected, minimal signal was visualized in the intestine of worms treated with only culture media (Fig. 3C and D). We therefore concluded that siRNA targeting Bma-LAD-2 transcript could enter the intestinal tract of adult filarial worms.

### Bma-LAD-2 siRNA treatment results in reduced transcript and protein expression.

**FIG 4:**
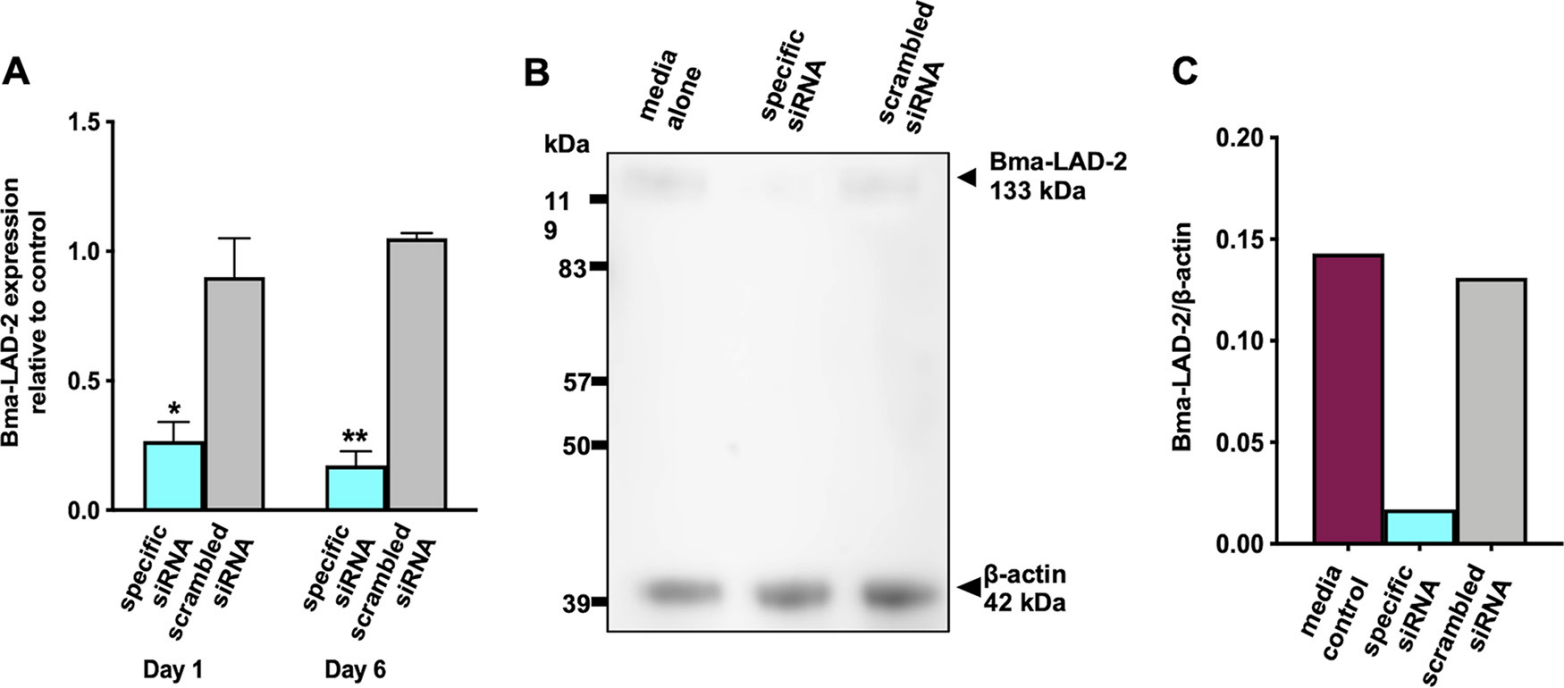
Treatment with Bma-LAD-2-specific siRNA reduces target transcript and protein levels in adult female B. malayi worms. Adult female filariae were cultured in Bma-LAD-2 siRNA, scrambled siRNA, or medium alone. (A) The *Bma-lad-2* transcript level was reduced in specific siRNA-treated groups (*n* = 3) compared to the scrambled controls (*n* = 3) normalized to *Bma-gapdh*. Ordinary one-way ANOVA followed by Tukey’s multiple-comparison test was used to generate the *P* values. These experiments were successfully repeated twice, and the data shown are from a single representative experiment, with means + SEM. (B) Bma-LAD-2 levels were assessed 24 h post-siRNA incubation by Western blotting using anti-Bma-LAD-2 antibodies. (C) Western blot quantification was performed using the ImageStudioLite software. The signal intensities of anti-Bma-LAD-2 were normalized to those of beta-actin.

After observation of Bma-LAD-2 siRNA entry into the intestine, gene and protein expression was evaluated by quantitative reverse transcription-PCR (RT-qPCR) and Western blotting, respectively. cDNA was generated using mRNA isolated from adult female B. malayi organisms cultured in medium alone, Bma-LAD-2 siRNA, and scrambled siRNA for 1 day and 6 days post-siRNA incubation. By quantifying B. malayi *lad-2* gene expression normalized to *Bma-gapdh* under each condition, we observed a 70.42% decrease in Bma-LAD-2 transcript levels (Fig. 4A) in worms treated with target-specific siRNA (mean, 0.2662) compared to the scrambled siRNA-treated filariae (mean, 0.9) relative to the medium control group at 1 day post-siRNA incubation. Bma-LAD-2 protein expression was visualized by immunoblotting in worms treated with specific or scrambled siRNA. A dramatic decrease in Bma-LAD-2 expression was observed in the specific siRNA-treated worms compared to the controls (Fig. 4B).

### Reduced worm viability and fecundity in Bma-LAD-2 siRNA-treated adult filariae.

**FIG 5:**
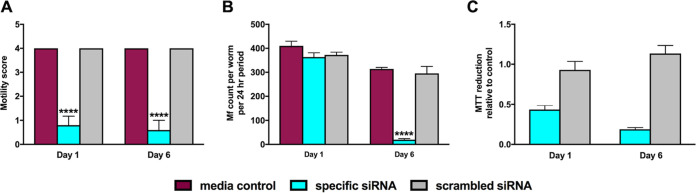
Bma-LAD-2 knockdown results in decreased worm motility, microfilaria release, and metabolism. Reducing Bma-LAD-2 expression in female B. malayi adult worms resulted in decreased motility (*n* = 5; day 1, *P* < 0.0001; day 6, *P* = 0.0004) (A), microfilaria count per worm (*n* = 5; day 1, *P* value not significant; day 6, *P* < 0.0001) per 24-h period (B), and metabolism (*n* = 2) (C) as measured by MTT reduction at 1 and 6 days post-siRNA treatment. For motility (A) and microfilaria release (B), an ordinary one-way ANOVA followed by Tukey’s multiple-comparison test was used to generate the *P* values. These experiments were successfully repeated twice, and the data presented are representative of a single experiment as means + SEM.

We evaluated the effects of Bma-LAD-2 knockdown on worm motility, Mf release, and (4,5-dimethylthiazol-2-yl)-2,5-diphenyltetrazolium bromide (MTT) reduction. At day 1 post-siRNA incubation, worm motility (Fig. 5A) was significantly reduced (*P* < 0.0001) in worms soaked in Bma-LAD-2 siRNA (mean, 0.8) compared to the control group (mean, 4). By day 6 postincubation, we observed an 85% reduction in motility relative to the controls (mean, 4) for the specific siRNA-treated group (mean, 0.6).

We next evaluated Mf release per adult worm per 24-h period for each group at 1 and 6 days post-siRNA incubation. While no reduction in Mf release was observed at 1 day after treatment with siRNA, Mf release was 93.4% lower from Bma-LAD-2 siRNA-treated adult filariae than Mf release from worms incubated with medium alone (Fig. 5B).

Finally, two randomly selected adult worms from each group were assessed by MTT reduction assay at each time point. At day 1 post-siRNA treatment, MTT reduction by B. malayi treated with target siRNA was 46% less than MTT reduction observed by worms treated with scrambled siRNA relative to the medium control group (Fig. 5C). By day 6, we observed an 83.25% decrease in MTT reduction by the Bma-LAD-2 siRNA group compared to worms treated with scrambled siRNA. Given the results described above, we conclude that Bma-LAD-2 is an essential protein for B. malayi adult worm survival *in vitro*, and knockdown results in death of the adult worm as defined by motility, fecundity, and metabolism.

### Bma-LAD-2 knockdown results in changes to the intestinal microvilli and mitochondria with loss of pseudocoelomic fluid.

**FIG 6:**
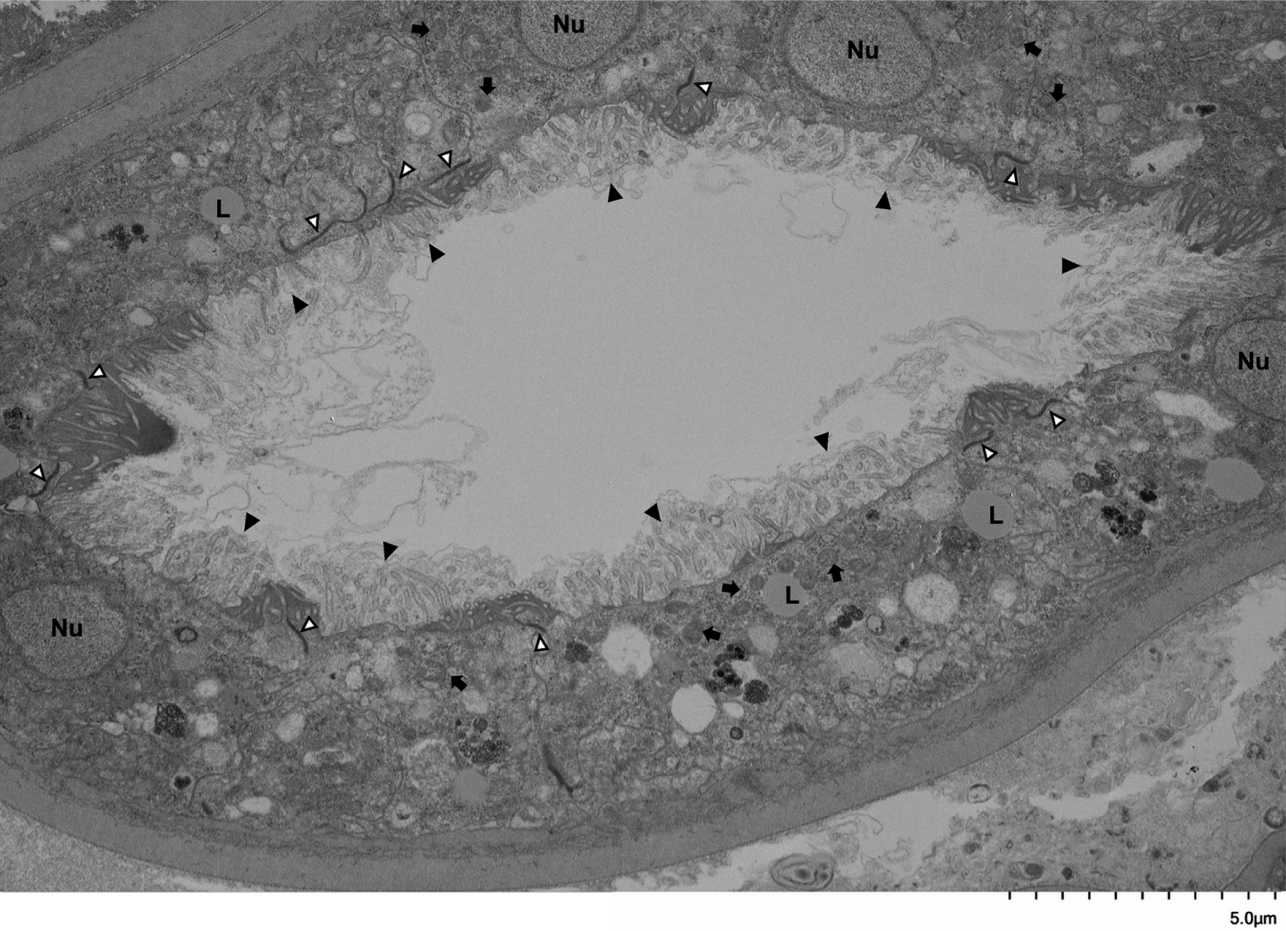
Intestinal tract of a female B. malayi adult worm. Image was captured by transmission electron microscopy (TEM) at 4,000×. Adult filariae were cultured in medium alone for 72 h. Microvilli (black arrowhead) line the apical surface of the intestinal epithelium. Other structures visible are apical junctions (white arrowhead), nuclei (Nu), lipid droplets (L), and mitochondria (black arrow).

**FIG 7:**
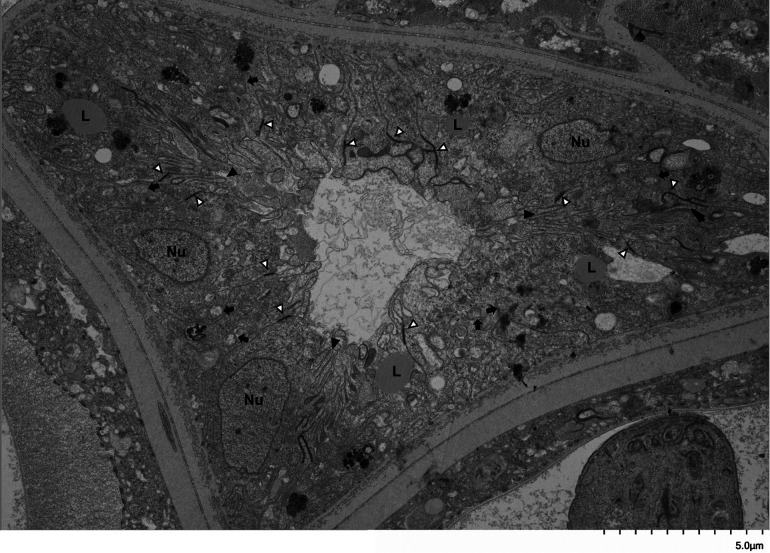
Intestinal tract of a female B. malayi adult worm treated with *Bma-*LAD-2 siRNA. Image was captured by transmission electron microscopy (TEM) at 4,000×. Adult filariae were incubated with *Bma-*LAD-2 siRNA for 24 h and then cultured in medium alone for an additional 48 h. Microvilli (black arrowhead) are largely absent from the apical surface of the intestinal epithelium. There are some vestigial microvilli that have been invaginated by the surrounding epithelium. Other structures visible are apical junctions (white arrowhead), nuclei (Nu), and lipid droplets (L). Many mitochondria (black arrow) appear to have unraveled cristae.

**FIG 8:**
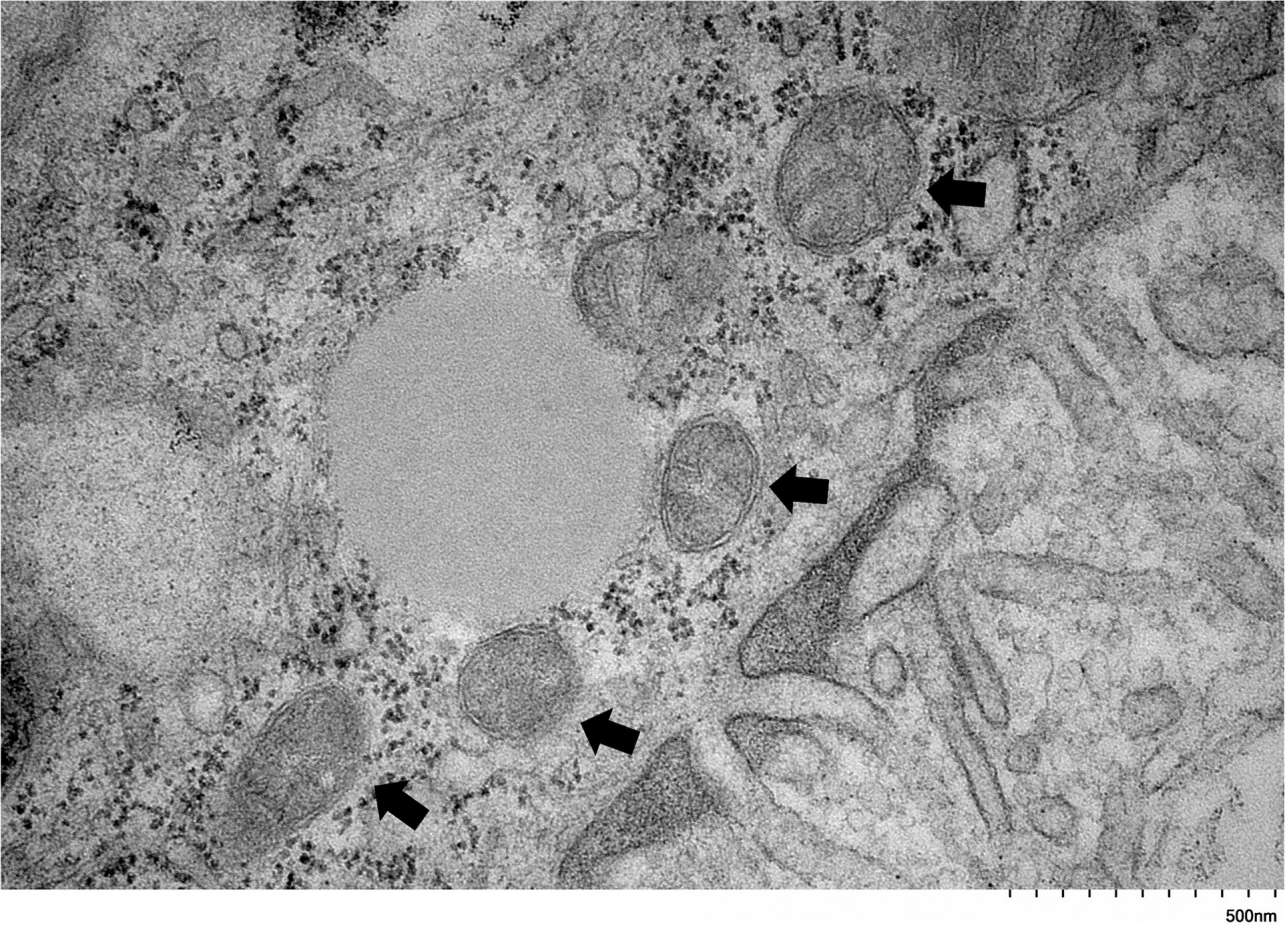
Transmission electron microscopy (TEM) image at 30,000× of mitochondria within intestinal epithelial cells of untreated control adult B. malayi worms. Mitochondria (black arrows) appear to be normal, with a continuous, folded cristae.

**FIG 9:**
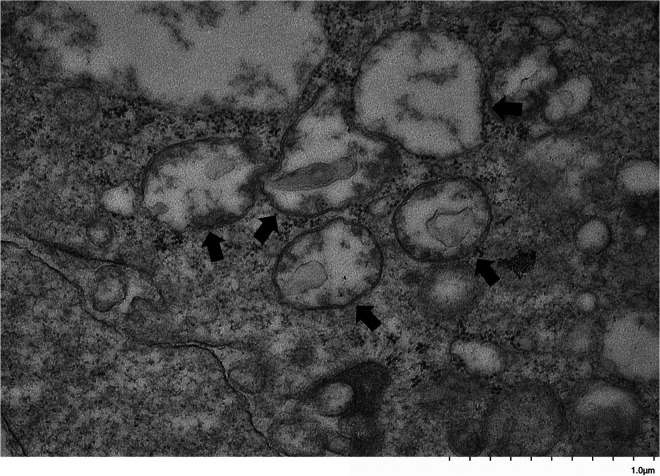
Transmission electron microscopy (TEM) image at 30,000× of mitochondria within intestinal epithelial cells of adult B. malayi worms after incubation with BMA-LAD-2 siRNA. Mitochondria (black arrows) appear to be diminished and have misshapen cristae.

Bma-LAD-2 is predicted to be an adhesion protein located at the apical junction of the intestinal tract (23). Therefore, we evaluated the structure of the adult filaria intestinal tract after treatment with Bma-LAD-2 siRNA using transmission electron microscopy (TEM). As seen in Fig. 6, microvilli lining the epithelium of the intestinal tract appeared present in untreated B. malayi adults. In contrast, adult worms treated with Bma-LAD-2 siRNA displayed a marked reduction in normal intestinal microvilli (Fig. 7). In addition, many of the apical junctions in the intestinal tract of treated worms appear shortened. To confirm this reduction in junctional length, we measured 38 junctions in both control and siRNA-treated worms (Data Set S1). On average, the junctions in treated filariae (mean, 636.81 nm) were significantly shorter than those of the controls (mean, 1,031.58 nm; *P* = 0.0004). A visual difference between the mitochondria of the treated and untreated worms was also observed. Compared to the controls (Fig. 8), many of the treated worms exhibited mitochondria with misshaped or diminished cristae (Fig. 9).

10.1128/mbio.03742-21.1 DATA SET S1 Measurements for apical junctions in the intestinal epithelium of treated and untreated filarial adult worms. Junction lengths were measured using ImageJ and converted into nanometers. A two-tailed Mann-Whitney test was performed to generate the *P* values. Download Data Set S1, XLSX file, 0.02 MB. Copyright © 2022 Flynn et al. 2022 Flynn et al. https://creativecommons.org/licenses/by/4.0/ This content is distributed under the terms of the Creative Commons Attribution 4.0 International license.

**FIG 10:**
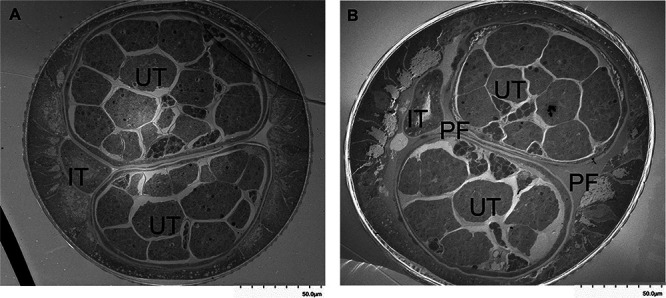
Adult B. malayi treated with *Bma-*LAD-2 siRNA exhibits loss of pseudocoelomic fluid. Cross-sectional images were captured by transmission electron microscopy (TEM) at 500×. Adult filaria treated with siRNA (A) exhibits loss of most of the pseudocoelomic fluid surrounding the intestinal and uterine tubes and appears to have fluid within the intestinal lumen. In contrast, the untreated filaria (B) has a normal distribution of pseudocoelomic fluid in the spaces around the intestine and uterine tracts. PF, pseudocoelomic fluid; IT, intestinal tube; UT, uterine tube.

Interestingly, when observed at lower magnification, enabling visualization of the entire nematode cross-section, it is apparent that the pseudocoelomic fluid is absent or markedly diminished in treated adult filariae (Fig. 10). While untreated worms display pseudocoelomic fluid separating the intestinal tract from the uterine tubes at all cross sections analyzed, Bma-LAD-2 siRNA-treated worms demonstrated direct contact between the intestinal tract and the uterine tubes. *In toto*, the imaging findings suggest that knockdown of Bma-LAD-2 results in reduction of tight junctions between intestinal epithelial cells and escape of pseudocoelomic fluid from the internal body cavity into the intestinal tract.

### No detectable Bma-LAD-2-specific IgG or IgE in serum from filarial patients.

A major concern when evaluating possible vaccine candidates for helminths is whether populations in areas of endemicity are IgE sensitized to the candidate antigen (31). Thus, we investigated whether serum from filaria-infected individuals contains IgE that recognizes Bma-LAD-2. A luciferase immunoprecipitation system assay was employed to detect antibody levels in the patient serum samples. The expression system produces a 3-dimensional recombinant Bma-LAD-2 protein fused to luciferase. Patients were categorized as presenting with asymptomatic microfilaremia (*n* = 13), chronic pathology (lymphedema) (*n* = 9), or tropical pulmonary eosinophilia (*n* = 8). Sera used in this experiment were obtained from patients prior to anthelmintic treatment. We also tested sera from individuals with no clinical or laboratory evidence of a filarial infection (*n* = 5) as well as healthy sera from blood bank donors (*n* = 5). As positive controls, we used affinity-purified polyclonal antibodies raised in rabbits immunized with recombinant Bma-LAD-2 as well as the rabbit antisera. Naïve rabbit sera served as a negative control.

**FIG 11:**
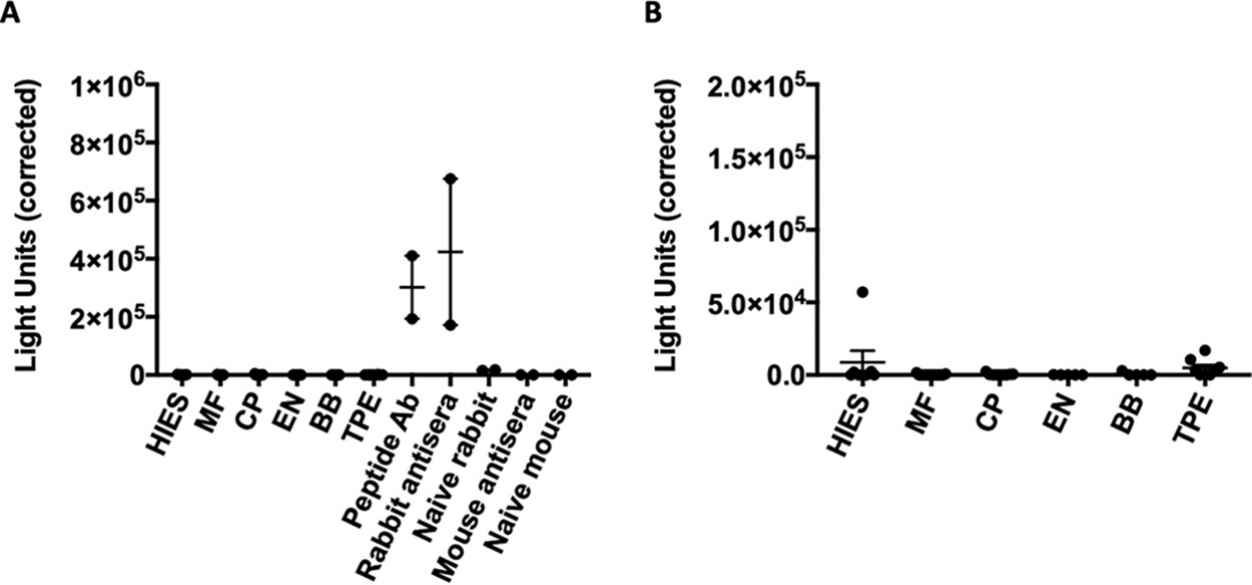
Filarial patient serum does not contain detectable IgG or IgE against Bma-LAD-2. Serum from filaria-infected individuals was incubated with a Bma-LAD-2-luciferase fusion protein. There was no detectable IgG (A) and IgE (B) in the patient serum as measured by the LIPS assay. As a positive control for IgG binding, Bma-LAD-2 rabbit polyclonal antibodies recognized the fusion protein. HIES, hyper-IgE syndrome; MF, microfilaremic; CP, chronic pathology; EN, endemic normal; BB, blood bank donors; TPE, tropical pulmonary eosinophilic; peptide Ab, Bma-LAD-2 rabbit polyclonal antibodies; mouse antisera, serum from *Litomosoides sigmodontis* vaccinated mice.

We found that serum samples from patients with lymphatic filariasis had no detectable preexisting IgG (Fig. 11A) or IgE (Fig. 11B) against Bma-LAD-2 fusion protein. As expected, there was recognition by the polyclonal antibodies and antisera against the fusion protein. This indicated that our fusion protein exhibited the proper conformation; thus, the absence of signal in filarial patient samples was due to absence of Bma-LAD-2-specific IgG or IgE.

### siRNA knockdown of other intestinal antigens of B. malayi.

In this study, we also attempted to evaluate whether 7 other intestinal proteins were essential for adult B. malayi survival (Table 1). The proteins selected for investigation were annotated as adhesion molecules, proteases, protease inhibitors, or involved in glycosylation based on work from previous studies (23, 30). We were able to successfully knock down gene expression for 3 of these target proteins. This limited success with siRNA inhibition was not entirely unexpected given the reported variability and difficulty of performing RNA interference in parasitic nematodes (32, 33). Of note, unlike knockdown of Bma-LAD-2, successful siRNA inhibition of Bma-serpin (a protease inhibitor) and Bma-peptidase (a protease) did not cause any appreciable phenotypic changes in adult B. malayi worms. siRNA inhibition of Bma-shtk, also a predicted protease, caused only a moderate decrease in metabolism with minimal effects on adult worm motility.

**TABLE 1:**
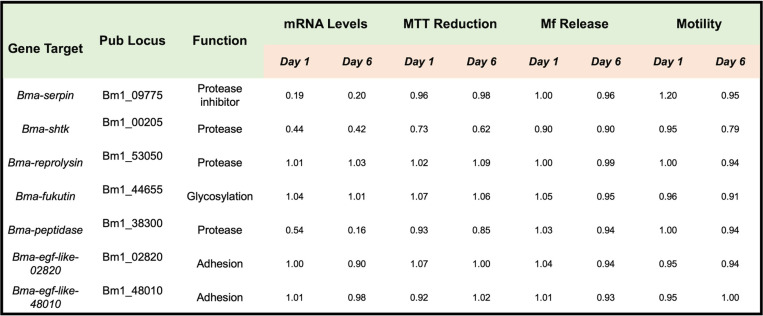
siRNA experiments targeting other intestinal proteins in B. malayi adult female worms^*a*^

a Values reported are relative to the controls, with a value of 1.00 indicating no difference. These siRNA experiments were only performed once.

## DISCUSSION

In this study, we sought to identify intestinal tract antigens of adult filarial nematodes that could serve as therapeutic or vaccine targets. Even though Bma-LAD-2 is expressed in all life cycle stages, we hypothesize that disruption of tight junctions formed within the intestinal tract of filariae will have a selective effect on the adult worm stages as microfilariae lack an intestinal tract (34). Knockdown of the tight junction protein Bma-LAD-2 caused rapid reductions in worm motility, metabolism, and microfilaria release, which led to worm death. Electron microscopy demonstrated a loss of pseudocoelomic fluid, revealing that disruption of the tight junctions between epithelial cells of the filarial intestinal tract could be a novel method to rapidly kill these worms by causing rapid dissolution of their hydrostatic skeleton.

Bma-LAD-2 is an immunoglobulin (Ig) intermediate-set (I-set) domain-containing protein and, therefore, belongs to the functionally diverse Ig superfamily (IgSF). The Ig domain is the basic structural unit of the superfamily and consists of two sandwiched antiparallel beta sheets (35). Proteins in the IgSF are classified based on the structure of their Ig domain and given a set designation of variable (V), constant 1 (C1), constant 2 (C2), or intermediate (I) (36). Ig I-set domains are similar to V-set domains but have a shorter distance between the invariant cysteine residues. InterPro analysis predicts that Bma-LAD-2 has 6 Ig domains spanning from amino acid position 24 to 602.

Based on homology to its ortholog in Caenorhabditis elegans (LAD-2, L1 adhesion), Bma-LAD-2 is predicted to be an L1 cell adhesion molecule (L1CAM). L1CAMs are single-transmembrane proteins that can participate in homophilic and heterophilic interactions (37). The cytoplasmic tail of L1CAMs has multiple consensus-binding sites, which allow for interaction with various cytoskeleton linker proteins, such as ankyrin, spectrin, and ERM (38, 39). Furthermore, the cytoplasmic tail of L1CAMs has phosphorylation sites, indicating a role in signal transduction (37, 40, 41).

Interestingly, in C. elegans, LAD-2 is critical in guiding axon migration and does not appear to be critical for the establishment or maintenance of the intestinal epithelium (42, 43). However, LAD-1, another L1CAM, has been shown to colocalize with apical junction molecules. In nematodes, cell adhesion molecules (CAMs) assemble to form two major types of apical junctions: the cadherin catenin complex (CCC) and the DLG-1/AJM-1 complex (38, 43). In C. elegans, it has been shown that the CCC is not essential for cell adhesion (44). This is surprising given the critical role of cadherins in cellular adhesion for most other eukaryotes. Researchers have suggested that LAD-1 acts as a redundant adhesion system, thereby mitigating the loss of the CCC. Indeed, embryos expressing dominant-negative LAD-1 have altered cell morphology and position, indicating a role in cellular adhesion (45–47). In filarial nematodes, no such redundancy appears to exist, as knockdown of Bma-LAD-2 resulted in dramatic phenotypic change.

Evidence also indicates that L1CAMs play an integral role in cell-to-cell contact signaling in the epithelial cells. In fact, loss of L1CAM signal can arrest cell proliferation and potentially induce apoptosis (48, 49). This would explain the ablation of microvilli in the intestine of target-specific siRNA-treated worms as well as the unraveling of the mitochondrial cristae. In addition, the loss of adhesion molecules may have hindered the ability of the apical junction to prevent diffusion of the internal pseudocoelomic fluid into the intestinal lumen. Loss of this fluid would have an adverse effect on worm vitality. The pseudocoelomic fluid generates a positive pressure within the pseudocoelom, creating a hydrostatic skeleton thought to be necessary for maintaining cuticle rigidity. This allows the longitudinal musculature of the worm to contract against the cuticle, creating the wave movement necessary for locomotion (34). In addition, it believed that the fluid serves as lubricant for the tissues during this motile process as well as a medium for nutrient exchange and cellular signaling (50). After an extensive search of the current literature, this loss of pseudocoelomic fluid appears to be a unique finding, and it most likely played a significant role in establishing the phenotype seen with Bma-LAD-2 knockdown.

In addition to disrupted anatomy, changes in the intestinal tract may have contributed to worm death by disrupting nutrient intake. While studies have shown that *Brugia* worms can absorb nucleotides, amino acids, small peptides, sugars, and vitamins through their cuticle (34, 51–55), a previous study of the rat filaria *Litomosoides sigmoiditis* showed the presence of red blood cells in the filarial gut, which implied that adult filariae actively feed (56). Another study demonstrated that heartworms are able to ingest serum (57). In addition, the proteomic analysis of different filarial tissue structures performed by our lab revealed that the filarial intestine is enriched in transporters, drug-metabolizing enzymes, proteases, protease inhibitors, and adhesion molecules (23). These findings suggest that the gut of adult filarial worms is used for not only nutrient digestion and uptake but also waste metabolism and disposal, functions essential in any living organism.

In addition to being present in adult B. malayi worms, Bma-LAD-2 is also found in the microfilariae and L3s. While these life cycle stages lack a functional intestinal tract (23, 34), it nonetheless would be important to test any medications or vaccines targeting Bma-LAD-2 for effects on these life cycle stages. The role of BMA-LAD-2 in these nonadult stages is unknown. Future studies may investigate the localization and function of this molecule in microfilariae and L3 stage worms.

The loss of Bma-LAD-2 results in a different phenotype than what has been observed when the ortholog of this protein is knocked out in C. elegans. This could be due to a number of reasons. C. elegans organisms constantly use their intestine for digestion and waste disposal, emptying their intestinal contents every 45 s (55, 58, 59). Additionally, *C elegans* cannot absorb nutrients across their thick cuticle, which leaves the intestine as the only means of nutrient absorption. Evolutionarily, it is reasonable that the systems in the intestine of C. elegans are redundant, as failure in one could result in worm death. Indeed, we see evidence of this redundancy by the fact that knocking out the CCC does not dramatically disrupt cell adhesion in C. elegans (44). In contrast, intestinal feeding by adult filariae may be more inconsistent as the parasites can absorb at least some nutrients through their cuticle (55, 58). It is quite possible that helminths only use their intestine to digest essential proteins and macromolecules too large to be absorbed by the cuticle. Consequently, there may have been less evolutionary pressure to develop redundant systems in the intestine. Finally, C. elegans is a free-living nematode; therefore, it has different digestive requirements than a parasitic nematode such as *Brugia*. This difference is no more apparent than with the number of protein-coding genes. C. elegans has 19,762 protein-coded genes compared to the predicted ~11,500 of B. malayi (30, 60).

While siRNA selection in our study was conducted to specifically target Bma-LAD-2, and while the siRNAs used did not exhibit homology to other B. malayi genes, an important limitation of this study is that we cannot completely rule out off-target effects of the siRNA. Another limitation to this study is that adult worms were obtained by overnight shipping after dissection of infected jirds. It is likely that the shipping caused some stress to the worms and may have decreased their baseline fecundity. Additionally, as reduction in fecundity in the setting of Bma-LAD-2 knockdown did not occur until reduction in worm metabolism, it is not possible to discern if Bma-LAD-2 plays a role in fecundity or if reduced fecundity simply occurred due to reduced worm viability. Evaluation of embryonic stages within adult gonads, which was not performed in this study, could better inform whether there are effects on embryogenesis.

When developing a helminth vaccine, there is a risk that the antigen could induce an allergic reaction in individuals previously exposed to filariae (22). This has been a major impediment to the development of effective helminth vaccines. There is evidence to suggest that helminth intestinal proteins act as “hidden antigens,” which are proteins not exposed to the immune system during natural infection and thus would not elicit an IgE response (17, 18, 22, 61, 62). Furthermore, because these proteins are hidden from the immune system, there is little evolutionary pressure to develop mechanisms that enable these proteins to evade the immune system. This may leave these proteins vulnerable to attack by the host defenses (62). A key limitation to development of vaccines against filarial nematodes is the possibility that populations that live where the disease is endemic are IgE sensitized against the antigen and thus experience allergic reactions when vaccinated. In this study, we demonstrated that people infected with lymphatic filariasis do not appear to have detectable IgE antibodies against Bma-LAD-2, suggesting that this antigen is safe to use as a vaccine. While this result is promising, more studies need to be performed using a larger sample size of people living where the disease is endemic to confirm these results.

In this study, we also evaluated 7 other *Brugia* intestinal proteins. Successful transcript knockdown was achieved for 3 of the targets. We attribute this failure rate to the well-documented difficulty of performing RNA interference in helminths (32, 33). None of the other proteins that were successfully knocked down (Bma-Serpin, Bma-ShTK, and Bma-Peptidase) resulted in substantial changes to worm viability or fecundity. It is expected that not all intestinal proteins are essential for adult filaria survival. Interestingly, inhibiting expression of Bma-Peptidase, a putative protease, did not result in a noticeable phenotype. This may be because there are multiple proteases present in the gut and that knockdown of more than one is necessary to affect worm survival.

Finally, we suspect that any therapeutics or vaccines developed against Bma-LAD-2 would be effective against *W. bancrofti* and B. timori as well as B. malayi due to high overall relatedness between the filaria species. In addition, Bma-LAD-2 shares a high level of homology (75%) with other filarial species. Therefore, a therapeutic or vaccine developed against this protein may be protective against other filarial infections.

In conclusion, we demonstrated knockdown of Bma-LAD-2 expression in adult B. malayi by siRNA inhibition. This resulted in ablation of microvilli, shortened tight junctions, unraveling of the mitochondrial cristae, and loss of pseudocoelmoic fluid, as visualized by TEM. We believe that these structural changes in the intestinal epithelium led to decreased worm motility, metabolism, and Mf release in worms treated with Bma-LAD-2 siRNA. Therefore, we conclude that Bma-LAD-2 is an essential protein for adult worm survival. The lack of Bma-LAD-2-specific IgE suggests that this antigen would be safe to use in a vaccine administered in areas of endemicity. In future studies we plan to evaluate Bma-LAD-2 as a vaccine candidate in animal models and as a potential therapeutic target for development of novel medications that specifically target adult filarial worms.

## MATERIALS AND METHODS

### Parasites and culture.

B. malayi female adults were obtained from the NIH/NIAID Filariasis Research Reagent Resource Center (FR3) and TRS Laboratories in Athens, GA. Adult worms were obtained by dissection of infected jirds 90 to 100 days postinfection and then shipped overnight to Uniformed Services University of the Health Sciences (USUHS). Before siRNA inhibition, adult worms were incubated in Dulbecco’s modified Eagle’s medium (Corning Cellgro) supplemented with 10% heat-inactivated fetal bovine serum (Atlanta Biologicals), 100 U/mL of penicillin, 100 μg/mL of streptomycin, and 1% l-glutamine (Sigma) for 24 h at 37°C in 5% CO_2_. Infection studies conducted at FR3 and TRS received approval from their respective Animal Care and Use Committees. Protocol approval for receipt of filarial worms from FR3 and TRS for use at the USUHS was granted by the USUHS Animal Care and Use Committee.

### Phylogenetic tree analysis.

We investigated the degree of relatedness between helminth orthologs and Bma-LAD-2. As an outlier group for the phylogenetic tree, we included dogs, humans, and cats, which were expected to have a significantly distant relation to Bma-LAD-2 given the low predicted sequence homology. The tree was constructed based on the likelihood estimation method for the LG model using aligned sequences by multiple-sequence comparison by log expectation (MUSCLE).

Orthologs from nematode species were selected using a BLAST query of the WormBase Parasite database (63) against the Bma-LAD-2 protein sequence (WBGene00227085). The following are the nematode species along with the accession codes for each ortholog identified: Brugia timori (BTMF_0000455001), Wuchereria bancrofti (maker-PairedContig_4689-snap-gene-5.23), Brugia pahangi (BPAG_0001424601), *Loa loa* (LOAG_18710), Dirofilaria immitis (nDi.2.2.2.t02266), *Haemonchus contortus* (HCON_00104790), Necator americanus (NECAME_12511), Onchocerca volvulus (Ovo-lad-2), Caenorhabditis elegans (lad-2), Acanthocheilonema viteae (nAv.1.0.1.t02543-RA), and *Ancylostoma duodenale* (ANCDUO_13310).

Orthologs from select mammals were identified using a BLAST query of the National Center of Biotechnology Information (NCBI) databases for the Bm-UGT peptide sequence. The following are the orthologs selected for analyses: Homo sapiens (GenBank accession no. NP_001153805.1), Canis lupus familiaris (XP_005640833.2), and Felis catus (XP_023103545.1).

### Structural analysis of Bma-LAD-2.

Molecular model of the monomer and dimer of the extracellular domain of Bma-LAD-2 (residues 19 to 1120) was generated based on available structures/oligomerization modes of homologous proteins. The Ig1-Ig4 region was modeled using the structure of neurofascin, a member of the L1 family of neural cell adhesion molecules (sequence identity of 31%, PDB entry 3P3Y) (25) and Ig5-6 and FN1-3 based upon the structures of contactin-3-6 (CNTN3-6), a group of glycophosphatidylinositol-anchored cell adhesion molecules (sequence identity of 28%, PDB entry 5I99) (64) and FN4-5 based upon the structure of a fragment encompassing the first four FN domains of the leukocyte common antigen-related protein (LAR), a postsynaptic type I transmembrane receptor protein (sequence identity of 27%, PDB entry 6TPW) (65). The dimer was assembled using the structure of neurofascin (PDB entries 3P3Y and 3P40) (25), which assembles into symmetrical dimers in the crystal. The figure was generated using the PyMOL Molecular Graphics System, version 2.0, from Schrödinger, LLC (https://pymol.org/2/).

### siRNA for RNAi.

BLOCK-iT RNA interference (RNAi) designer was employed for selecting siRNA duplexes of candidate genes for gene silencing activity and specificity. The siRNA sequence with the greatest probability of success was selected for each target, and for some targets multiple sequences were selected to improve knockdown success. In addition, the siRNA sequences were then evaluated for homology with other mRNA sequences from the B. malayi transcriptome database in WormBase and NCBI to limit off-target knockdown. None of the siRNA sequences shared homology with mRNA sequences outside those selected for knockdown. Life Technologies synthesized the target-specific siRNAs for Bma-LAD-2, Bma-Fukutin, Bma-ShTK, and Bma-Serpin and purified the complexes by standard desalting methods. Target-specific siRNAs for Bma-EGF-like-02820, Bma-EGF-like-48010, Bma-Peptidase, and Bma-Reprolysin were obtained through Dharmacon. The 5′ to 3′ siRNA sequences used in this experiment are the following: Bma-LAD-2 siRNA 1, sense, 5′ GCAAGUACUACCAUACUAUdTdT 3′; antisense, 5′ AUAAGUUGGAAUUCGUUGCdTdT 3′; Bma-LAD-2 siRNA 2, sense, 5′ GCGCAUAUCGCAAGUAAAUdTdT 3′; antisense, 5′ AUUUACUUGCGAUAUGCGCdTdT 3′; Bma-LAD-2 siRNA 3, sense, 5′ GCGAAUAGUCGAUACCUAAdTdT 3′; antisense, 5′ UUAGGUAUCGACUAUUCGCdTdT 3′; Bma-Fukutin siRNA 1, sense, 5′-CCACCCATTTCGCAGATTT-3′; antisense, 5′ AAAUCUGCGAAAUGGGUGGdTdT 3′; Bma-Fukutin siRNA 2, sense, 5′ GGAGCGAGAGTGAATGGAAdTdT 3′; antisense, 5′ UUCCAUUCACUCUCGCUCCdTdT 3′; Bma-Fukutin siRNA 3, sense, 5′ GCTAACGTTGCAAATTATTdTdT 3′; antisense, 5′ AAUAAUUUGCAACGUUAGCdTdT 3′; Bma-ShTK siRNA 1, sense, 5′ GCGCCTTCTACAGCAGTAAdTdT 3′; antisense, 5′ GCGCCUUCUACAGCAGUAAdTdT 3′; Bma-ShTK siRNA 2, sense, 5′ GGUGGUAUGAAUAGCAUAAdTdT 3′; antisense, 5′ UUAUGCUAUUCAUACCACCdTdT 3′; Bma-ShTK siRNA 3, sense, 5′ GCUAAAGAACUAUGCGCUAdTdT 3′; antisense, 5′ UAGCGCAUAGUUCUUUAGCdTdT 3′; Bma-Serpin siRNA, sense, 5′ GGAUUUCGAGUGAGACAAAdTdT 3′; antisense, 5′ UUUGUCUCACUCGAAAUCCdTdT 3′; Bma-EGF-like-02820 siRNA, sense, 5′ GUAUCGAGGGCAAGGGAAAdTdT 3′; antisense, 5′ UUUCCCUUGCCCUCGAUACdTdT 3′; Bma-EGF-like-48010 siRNA, sense, 5′ GCAACAAAUGCAAGAAUAAdTdT 3′; antisense, 5′ UUAUUCUUGCAUUUGUUGCdTdT 3′; Bma-Peptidase siRNA 1, sense, 5′ AGGAAAGGUUGUUAGGAUAdTdT 3′; antisense, 5′ UAUCCUAACAACCUUUCCUdTdT 3′; Bma-Reprolysin siRNA 3, sense, 5′ GGAUAAUGUGAAAGGAAUAdTdT 3′; antisense, 5′ UAUUCCUUUCACAUUAUCCdTdT 3′.

### Assessment of siRNA uptake by fluorescence microscopy.

Adult female worms were incubated with 5 μM 5′ cy3-labeled *Bma-lad-2* siRNA 1 (Sigma-Aldrich) for 24 h to evaluate uptake of siRNA into intestinal tract epithelial cells. Adult female worms were cultured in medium alone as a negative control. As a counterstain, samples were treated with 10 μg/mL 4′,6-diamidino-2-phenylindole dihydrochloride (DAPI; Sigma-Aldrich) in phosphate-buffered saline (PBS). Images were obtained with a Nikon Eclipse E600 fluorescence microscope and converged by NIS-Elements software.

### siRNA treatment of B. malayi.

For siRNA inhibition of the target gene expression, we soaked B. malayi adult female worms in culture media with siRNA, slightly modifying a previously established protocol (66). For each time point, we incubated 5 adult female worms for 24 h in an equal mixture of the siRNAs (Bma-LAD-2, Bma-Fukutin, Bma-ShTK, and Bma-Serpin) or one siRNA (Bma-EGF-like-02820, Bma-EGF-like-48010, Bma-Peptidase, and Bma-Reprolysin) at a total concentration of 5 μM in 850 μL of medium in a 5,000 molecular-weight-cutoff Pur-A-Lyzer dialysis tube (Sigma-Aldrich). Previous studies have shown that this concentration of siRNA sufficiently silences gene expression (66–69). We placed the dialysis tubes in a beaker with 500 mL of culture medium at 37°C in 5% CO_2_. For the control groups, 5 adult female worms were incubated alone in media or with scrambled siRNA (5 μM) under conditions similar to those for the target siRNA-treated group. The worms were extracted after the 24-h incubation and placed individually into 1 mL of culture media. Initially, worms were evaluated 1 day postincubation for transcript knockdown, worm motility, MTT reduction, and microfilaria release. For Bma-LAD-2, we conducted additional experiments to evaluate the worms at 6 days post-siRNA incubation.

### Evaluation of worm motility.

Motility was evaluated based on the following scale: 4, active movement; 3, modest reduction in movement; 2, severe reduction in movement; 1, twitching; and 0, no movement. A blinded observer rated worm motility for each group under a dissecting microscope.

### Measuring MTT reduction.

Metabolic function was evaluated using a (4,5-dimethylthiazol-2-yl)-2,5-diphenyltetrazolium bromide (MTT) assay from Sigma (70). For each group per time point, 2 randomly selected worms were treated with 0.5 mg/mL MTT in 0.5 mL of PBS for 30 min at 37°C in 5% CO_2_. Each worm was then transferred into a well containing 200 μL of DMSO of a 96-well plate at room temperature for 1 h. Quantification of MTT reduction was measured based on absorbance relative to a DMSO blank at 570 nm with a Synergy HTX multimode plate reader (BioTek).

### Quantifying Mf release.

Prior to quantifying Mf release, we incubated the adult worms in 1 mL of fresh media for 24 h. The adult filariae were then removed for evaluation by the MTT reduction assay and RT-qPCR. The Mf were enumerated under a light microscope at high magnification in the wells containing expended media.

### RNA extraction and analysis of RNA levels by RT-qPCR.

Adult B. malayi female worms were treated with TRIzol (Thermo Fisher Scientific) and subjected to three freeze/thaw cycles. Adult filariae were then placed in Matrix D lysis tubes (MP Biomedicals) and homogenized by a FastPrep-24 Biopulverizer (MP Biomedicals) for 7 min at 6 m/s. We added chloroform to the homogenate and phase separated the mixtures in Phase Lock gel tubes (5Prime) at 11,900 × *g* for 15 min at 4°C. After the top layer (aqueous phase) was collected, we precipitated the RNA using cold isopropanol and then pelleted it at 12,000 × *g* for 1 h. The RNA pellet was washed twice using cold 75% ethanol. We resuspended the RNA in nuclease-free water and quantified the sample concentrations using a NanoDrop 1000 (Thermo Fisher Scientific). Using Superscript IV (Thermo Fisher Scientific), we synthesized cDNA from mRNA per the manufacturer’s protocol. We quantified target gene and B. malayi housekeeping gene *gapdh* expression levels in duplicate 20-μL reaction mixtures using 1 μL of 20× TaqMan gene expression assay (Thermo Fisher Scientific), 1 μL of cDNA, and 18 μL of TaqMan gene expression master mix (Applied Biosystems). We employed the following PCR conditions with a 7500 real-time PCR system (Applied Biosystems): 2 min at 50°C, 10 min at 95°C, and 40 cycles of 15 s at 95°C and 1 min at 60°C cycle of 50°C. The following TaqMan primer and internal probes were used: *Bma-gapdh*, forward primer, 5′-TTGATCTCACTTGCCGACTC-3′; reverse primer, 5′-TGGTCTTCGGTGTATTCCAA-3′; internal probe, 5′-CAGCTAATGGACCGATGAAGGGGA-3′; *Bma-lad-2*, forward primer: 5′-GTGATCCACGGCTTACGATT-3′; reverse primer: 5′-CAGGCACATCAAGCACAGTT-3′; internal probe: 5′-TGCTCGTGGCTTTCATTCAGGA-3′; *Bma-futkin*, forward primer, 5′-AGGTTATTTCATGTGCCCTGC-3′; reverse primer, 5′-ATTCCATTCACTCTCGCTCCA-3′; internal probe, 5′-AGGCGGATTACGGTAATTGGCGAGT-3′; *Bma-shtk*, forward primer, 5′-TGCACTGATCCAATGGCAAA-3′; reverse primer, 5′-GTTACTGCTGTAGAAGGCGC-3′; internal probe, 5′-TGCGCCAAAACATGTGGATTTTGCGG-3′; *Bma-serpin*, forward primer, 5′-ACGTGCGCAGTTAGACTTTG-3′; reverse primer, 5′-GCCTCTGCGATATAAGCCAA-3′; internal probe, 5′-GCGGACGGTGAAACGAAGCAGCA-3′; *Bma-egf-like-02820*, forward primer, 5′-GCTTACACGGTGGCAGAAAA-3′; reverse primer, 5′-AAGCCACCTATCTGCTCTCC-3′; internal probe, 5′-TCGAGGGCAAGGGAAAACTGGAA-3′; *Bma-egf-like-48010*, forward primer, 5′-ACCTGGCTTCATGGGAGAAA-3′; reverse primer, 5′-CTTCACCACAGTCGCAAACA-3′; internal probe, 5′-TGCTGCCGGTCTTATGGGCG-3′; *Bma-peptidase*, forward primer, 5′-CAGCCATTATTGGCCAGGAC-3′; reverse primer, 5′-AAATGAAGTGGTGCCGCATT-3′; internal probe, 5′-AGCCTTCCAACTTGGTTCATCCCAACA-3′; *Bma-reprolysin*, forward primer, 5′-TGGAACACAGTGATCAGGCT-3′; reverse primer, 5′-AACGGCATTCCACTTATCG-3′; internal probe, 5′-CCCATTTCGTGTGCAATAGTTGCAGCA-3′.

### Generation of anti-Bma-LAD-2 polyclonal antibodies.

For the immunoblot analysis and luciferase immunoprecipitation system (LIPS) assay, polyclonal anti-Bma-LAD-2 peptide antibodies were generated by GenScript. Rabbits were immunized against nearly full-length Bma-LAD-2 peptide (21 to 1,120 aa).

### Immunoblot analysis of Bma-LAD-2.

Prior to Western blot analysis, we incubated B. malayi adult female worms in 5 μM Bma-LAD-2 siRNA for 24 h, followed by an additional 24-h culture in fresh media. The adult filariae were transferred into Matrix D lysis tubes (MP Biomedicals) with PBS (pH 7.4) and 4 μL of Halt protease inhibitor cocktail (Thermo Scientific) and then homogenized using a FastPrep-24 biopulverizer (MP Biomedicals) for 3 min at 4 m/s. Protein concentration was quantified by Bradford protein assay (Bio-Rad). Protein lysate (10 μg) was separated on 10% Bis-Tris NuPAGE gel (Invitrogen) and then transferred onto 0.2-μm nitrocellulose filter paper (Bio-Rad). The filter paper was blocked overnight in 5% bovine serum albumin (BSA) in Tris-buffered saline with 0.1% Tween 20 (TBS-T). After the overnight blocking, the membrane was incubated with 1:7,000 polyclonal anti-Bma-LAD-2 peptide antibodies (GenScript) and 1:1,000 rabbit anti-β actin antibodies (Abcam) for 1 h. The membrane was washed three times with TBS-T for 15 min. Horseradish peroxidase-conjugated goat anti-rabbit IgG antibody was incubated for 1 h with the filter paper at a dilution of 1:2,000. After washing again with TBS-T, the membrane was developed with the chemiluminescent reagent SuperSignal West Pico plus (Thermo Scientific).

### Transmission electron microscopy.

B. malayi female worms (3) were treated with Bma-LAD-2 siRNA for 24 h and then cultured for an additional 48 h. An equal number of adult female worms was incubated in medium alone for the same amount of time. Both groups of filariae were processed for imaging by electron microscopy. This whole process was repeated on two separate occasions. For morphological evaluation, the female filariae were first washed in PBS (pH 7.4) and then fixed with 2.5% paraformaldehyde, 1% glutaraldehyde in 0.12 M Millong’s phosphate buffer (pH 7.4) overnight at room temperature. Following this step, the samples were postfixed with 1% osmium tetroxide in 0.12 M Millong’s phosphate buffer (pH 7.4) for 100 min and then fixed en bloc with 2% aqueous uranyl acetate for 90 min. The samples were dehydrated in graded ethanol solutions (75% to 100%) for 10 min each. The worms were embedded in low-viscosity epoxy resin (modified Spurr’s recipe) and then dried at 70°C overnight. Ultrathin sections (75 nm) were cut on a Reichert Ultracut E Ultramicrotome and then stained with 0.2% lead citrate. Reagents used were obtained from Electron Microscopy Services. Samples were visualized using a Hitachi HT7700 transmission electron microscope at an accelerating voltage of 80 kV.

Apical junctions were measured primarily using ImageJ because of their curved nature (71). Using the EM software, 8 junctions were measured in nanometers. A conversion factor was generated by dividing these 8 EM measurements by their respective ImageJ values and then calculating the mean. This conversion factor was then applied to all the ImageJ values to obtain their estimated lengths, in nanometers. For both the treated and untreated groups, 38 apical junctions were measured.

### Ruc-antigen fusion protein.

The Bma-LAD-2-*Renilla reniformis* luciferase (Ruc) construct was inserted into a pREN2 vector by GenScript. The predicted signal sequence was removed prior to gene synthesis. The vector was cloned into TOP10 cells (Thermo Fisher Scientific) and amplified on agarose plates with kanamycin at 50 μg/mL. Plasmid DNA was isolated and purified using a Miniprep kit (Qiagen) per the manufacturer’s guidelines. 293F cells (Thermo Fisher Scientific) were transfected with 30 μg of Bma-LAD-2 plasmid at a concentration of 1 × 10^6^ cells per mL. The 293F cells were collected 72 h later and homogenized. The lysate was stored at −80°C for later use.

### LIPS.

We employed the LIPS assay to measure antibody titers in serum from *W. bancrofti*-infected patients (72–74). In a 96-well polypropylene plate, serum was diluted to 1:100 for IgG and 1:10 for IgE in 50 μL of LIPS master mix (20 mM Tris, pH 7.5, 150 mM NaCl, 5 mM MgCl_2_, 1% Triton X-100) with PBS-Tween 20 added to bring the volume to 100 μL. We added 1 × 10^6^ light units (LU) of the LAD-2-Ruc fusion protein to the mixture and incubated it at room temperature for 10 min. Purification of the antigen-antibody complex involved adding 5 μL of a 50% suspension of Ultralink protein A/G (Pierce) or Ultralink anti-human IgE beads in PBS to a 96-well filter plate (Millipore) and then applying a vacuum. The serum mixture was then added and allowed to incubate for 15 min at room temperature. A vacuum was applied to the filter plate, leaving only antigen-antibody complex bound to beads in the wells. The samples were washed with 200 μL of LIPS master mix twice and with PBS once. Using a Bethold LB 960 Centro microplate luminometer, emitted LUs were measured after addition of 50 μL of coelenterzine solution (Promega) to each sample well. The serum samples were run in duplicate, and the calculated LU was the emitted LU for only the LAD-2 fusion protein subtracted from the emitted LU for each sample.

### Serum samples.

Serum samples used in this study were obtained under Institutional Review Board (IRB)-approved protocols from the Department of Transfusion Medicine (Clinical Center, National Institutes of Health, Bethesda, MD) and from the Laboratory of Laboratory of Parasitic Diseases (NIAID, National Institutes of Health, Bethesda, MD). All donors provided written approved consent.

### Statistical analysis.

The siRNA experiments for Bma-LAD-2 were repeated twice under the same conditions. All other siRNA experiments were only performed once. The worm motility and Mf release data were analyzed by one-way analysis of variance (ANOVA) using the statistical package in PRISM 7.0. Validity of the one-way ANOVA was verified by performing individual comparisons of mean values using Tukey’s multiple-comparison test. For the gene expression and MTT reduction data, a *t* test was used to determine significance. For the junctional measurements, a two-tailed Mann-Whitney test was performed. The *P* values for each experimental and control group was designated the following: *, *P* < 0.05; **, *P* < 0.01; and ***, *P* < 0.001.

### Data availability.

All relevant data are within the paper and the supplemental material.
