# Supplementary figures and images for: Bma-LAD-2, an Intestinal Cell Adhesion Protein, as a Potential Therapeutic Target for Lymphatic Filariasis
Source: mBio. 2022 Apr 27;13(3):e03742-21. doi: 10.1128/mbio.03742-21 (PMC9239158; doi:10.1128/mbio.03742-21)

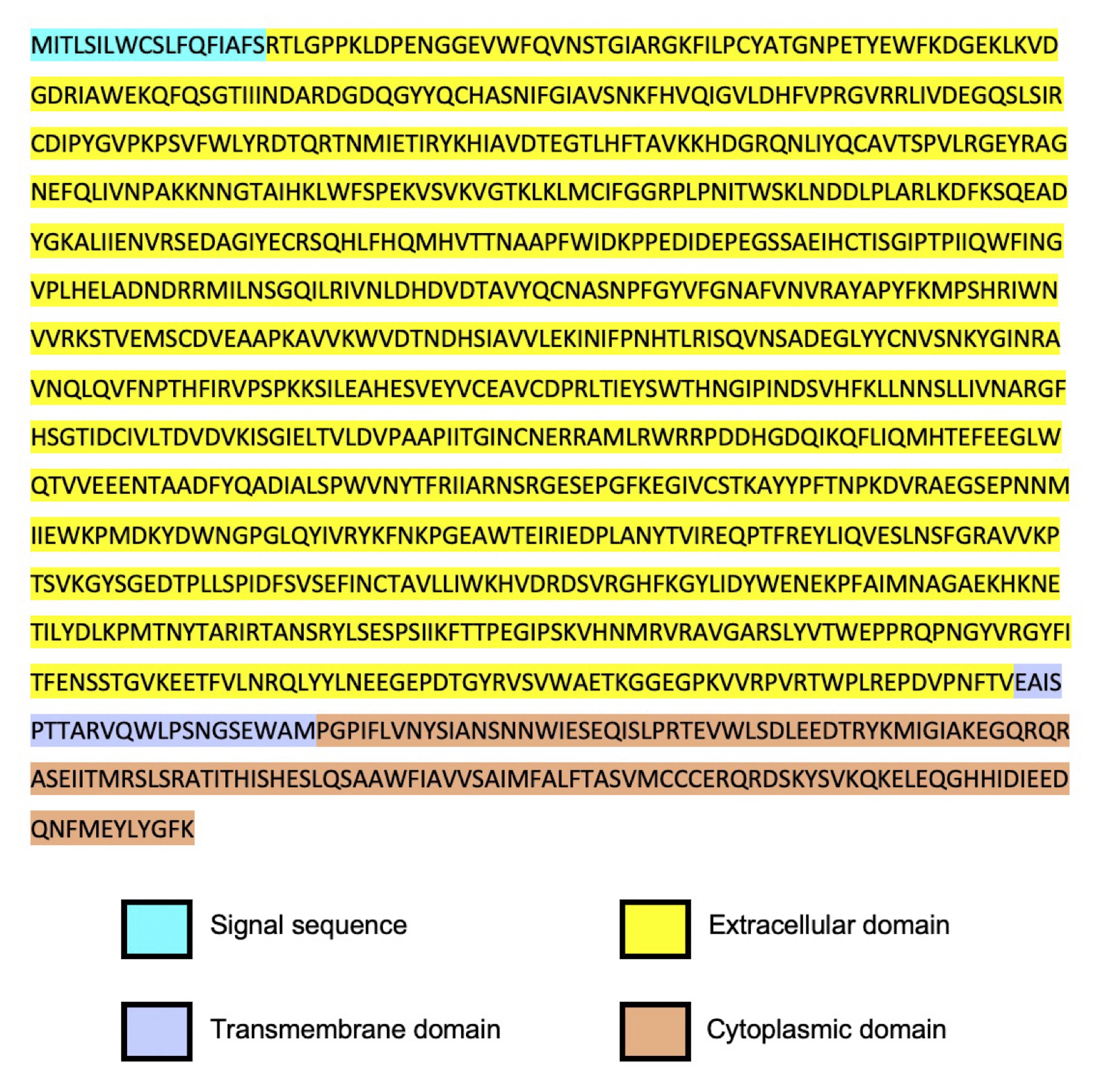

Supplement: FIG S1 [file mbio.03742-21-s0001.jpg]
